# Transcriptome-wide association study for postpartum depression implicates altered B-cell activation and insulin resistance

**DOI:** 10.1038/s41380-022-01525-7

**Published:** 2022-04-01

**Authors:** Jerry Guintivano, Karolina A. Aberg, Shaunna L. Clark, David R. Rubinow, Patrick F. Sullivan, Samantha Meltzer-Brody, Edwin J. C. G. van den Oord

**Affiliations:** 1 Department of Psychiatry, University of North Carolina at Chapel Hill, Chapel Hill, NC USA; 2 Center for Biomarker Research and Precision Medicine, Virginia Commonwealth University, Richmond, VA USA; 3 Department of Psychiatry & Behavioral Sciences, Texas A&M University, College Station, TX USA; 4 Department of Genetics, University of North Carolina at Chapel Hill, Chapel Hill, NC USA; 5 Department of Medical Epidemiology and Biostatistics, Karolinska Institutet, Stockholm, Sweden

**Keywords:** Genetics, Molecular biology, Depression

## Abstract

Postpartum depression (PPD) affects 1 in 7 women and has negative mental health consequences for both mother and child. However, the precise biological mechanisms behind the disorder are unknown. Therefore, we performed the largest transcriptome-wide association study (TWAS) for PPD (482 cases, 859 controls) to date using RNA-sequencing in whole blood and deconvoluted cell types. No transcriptional changes were observed in whole blood. B-cells showed a majority of transcriptome-wide significant results (891 transcripts representing 789 genes) with pathway analyses implicating altered B-cell activation and insulin resistance. Integration of other data types revealed cell type-specific DNA methylation loci and disease-associated eQTLs (deQTLs), but not hormones/neuropeptides (estradiol, progesterone, oxytocin, BDNF), serve as regulators for part of the transcriptional differences between cases and controls. Further, deQTLs were enriched for several brain region-specific eQTLs, but no overlap with MDD risk loci was observed. Altogether, our results constitute a convergence of evidence for pathways most affected in PPD with data across different biological mechanisms.

## Introduction

Postpartum depression (PPD), a diagnostic subtype of major depressive disorder (MDD) that occurs in the postpartum period, is a common complication of the perinatal period. It affects approximately half a million women annually in the U.S [1–4] and is one of the greatest causes of maternal morbidity and mortality [5, 6]. Additionally, PPD can have long-term adverse consequences on the newborn [7–11]. Despite this impact to public health, there is a lack of studies investigating the biology behind PPD. Although the precise mechanisms are unknown, PPD is a complex disorder that likely involves the culmination of genetic risk factors, response to hormonal fluctuations, and environmental factors.

Pregnancy is characterized by dynamic physiological changes that are expected to return to pre-pregnancy levels during the postpartum period. The stress axis, reproductive system, glucoregulation, and immune activation are a few examples of biological systems that interact and adapt to support the growing fetus. Perturbations in the recovery of these systems after childbirth, in addition to other risk factors, could result in PPD symptoms. Performing transcriptome-wide association studies (TWAS) allows for the interrogation of functional changes associated with case status. Employing TWAS, we can identify the biologically relevant changes in PPD that result from the aforementioned system disruptions, providing crucial insight into specific causes of the disorder.

Traditionally, TWAS have been performed on bulk tissues that are composed of multiple diverse cell types. This cellular heterogeneity has a detrimental impact on the ability to detect disease associations [12]. Thus, in bulk tissue, case-control differences will be “diluted” when they affect only one or few cell types, may cancel out if the differences are of opposite signs across cell types, or may be undetectable altogether if the differences involve low abundant cells. Furthermore, identifying the specific cell types from which the association signals originate is key to formulate refined hypotheses of PPD disease pathology, designing proper follow-up experiments, and to develop effective clinical interventions. Efforts to address these issues with cell type-specific effects have been attempted using purified cell populations or single-cell RNA sequencing. However, these methods are labor-intensive and/or cost-prohibitive for most large-scale transcriptomic interrogation. As an alternative, statistical methods have been developed to deconvolute effects of individual cell types using data generated from bulk tissue [12–17].

Transcriptomic information can also be combined with other data types leading to deeper mechanistic insight into the regulation of transcription. For example, single nucleotide polymorphisms (SNPs) have been shown to regulate expression in a cell type-specific manner [18, 19]. In addition, DNA methylation can regulate gene transcription [20, 21]. Identifying the (epi-) genomic regulators of transcriptional differences is a key step for generating novel hypotheses about PPD disease etiology. This would allow, for example, designing functional follow-up studies. The identification of (epi-) genomic regulators also has translational value as they are potential targets for correcting aberrant transcription.

In this work, we performed the largest TWAS for PPD, using RNA-sequencing of whole blood, in a cohort of women six-weeks following childbirth. To date, three TWAS studies have been performed with sample sizes ranging from 6 to 15 cases and 10 to 122 controls [22–24]. Further, the analyses presented here are performed on a cell type-specific level. Additionally, SNPs, DNA methylation, and hormone levels were used to identify regulators underlying the observed case-control transcriptional differences. Altogether, this represents the largest and most diverse interrogation into the biology of PPD.

## Results

We recruited a case-control cohort of 1551 women (579 PPD cases, 972 controls) at six weeks postpartum with PPD case-control status established using clinical interviews. Participants were racially (66.5% Black, 32.9% White, and 0.6% Asian) and ethnically (15.9% Hispanic) diverse (Table S1) [25]. We generated whole blood-derived transcription data using RNA-sequencing (RNA-seq), which resulted in 134,302 known transcripts from 51,079 genes (88.2% of all Ensembl annotated genes) [26]. In addition, to study regulators of PPD expression differences we generated array-based DNA methylation data (Illumina 450k), assaying 373,635 CpGs, and genome-wide SNP data which, with imputation, assayed ~12.5 million variants. Cell type proportions were estimated from DNA methylation data using standard methods [14, 27]. The average estimated cell type proportions were 9.7% CD8^+^ T-cells, 16.3% CD4^+^ T-cells, 5.6% B-cells, 4.7% monocytes, 59.4% granulocytes, and 3.0% natural killer cells. The validity of the estimated proportions was established through high correlations with complete blood counts available for a subset of participants (Supplemental Results) [14, 27]. We observe differences in cell proportions between cases and controls for CD4^+^ T-cells (*β* = −0.06, *p* = 0.03) and granulocytes (*β* = 0.06, *p* = 0.02), but not for the other cell types (Table S1). All downstream analyses control for cell type proportions.

### TWAS for PPD identifies cell type-specific dysregulation

**Fig. 1:**
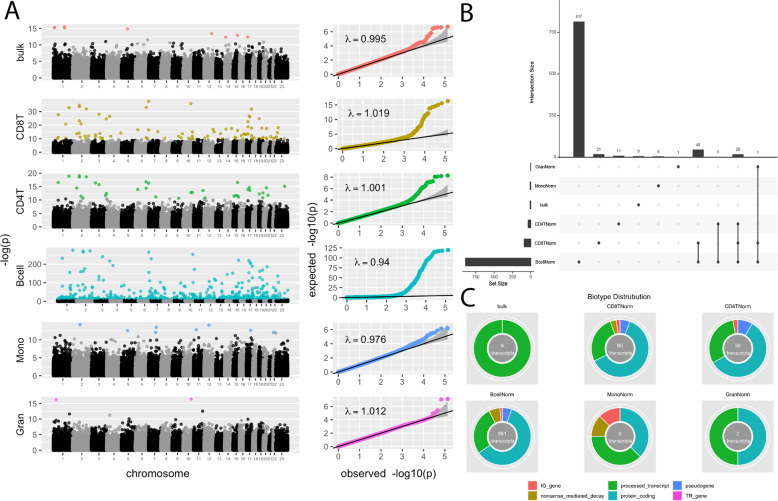
PPD TWAS cell type-specific associations. **A** Manhattan plots and QQ-plots of PPD transcriptome-wide association study (TWAS) results in bulk tissue and individual cell types. Non-significant transcripts are shown in grey/black. Significant transcripts at an FDR < 0.1 are shown in color. **B** Upset plot showing overlap of significant TWAS transcripts across bulk tissue and individual cell types. **C** Biotype distribution of significant TWAS transcripts in bulk tissue and individual cell types.

In addition to TWAS of whole blood, we performed cell-type-specific TWAS using a statistical deconvolution approach [12, 16, 18, 28, 29]. Figure 1A shows Manhattan plots, QQ-plots and lambdas for the TWAS results for whole blood and each cell type separately. Across all analyses, we correct for multiple testing using false discovery rate (FDR), with a q-value < 0.1 threshold indicating significance. Decreasing the q-value to 0.05 would, for example, result in only a modest reduction in false positives but decreases the probability of finding true effects exponentially as shown previously [30]. The TWAS in whole blood and in the different cell types each revealed transcripts that were significantly associated with PPD (see Tables S2–S7 for all transcripts with p-value < 0.05). The QQ-plots and lambdas from the TWAS results (Fig. 1A) in combination with TWAS of permuted case-control status for each analysis (Fig. S1), which yielded average lambdas of approximately one, indicated no evidence of test-statistic inflation.

#### Whole blood

TWAS in whole blood revealed nine transcripts from nine genes that were significantly associated with PPD (Table S2). The most significant transcript was for the small nuclear RNA, *RNVU1-9* (*p*-value = 1.92 × 10^−7^). Using the Ensembl biotype annotations [26], which provide an indication of transcript function, we see that all nine transcripts are processed transcripts (Fig. 1C).

#### Granulocytes

TWAS in granulocytes had two transcripts from two genes significantly associated with PPD case status: a protein-coding transcript for *SH3PXD2A* (*p*-value = 8.81 × 10^−8^) and a processed transcript for *CSF3R* (*p*-value = 7.45 × 10^−8^) (Fig. 1C; Table S3).

#### Monocytes

Monocyte TWAS resulted in eight transcripts from eight genes that reached transcriptome-wide significance (Table S4). Transcript biotypes are varied with three protein-coding, three processed transcripts, one nonsense-mediated decay, and one immunoglobulin (IG) gene (Fig. 1C). The top hit (*p*-value = 6.60 × 10^−7^) is a protein-coding transcript for the gene *VWA3B*.

#### CD8^±^ T-cells

There were 90 transcripts in 87 genes that reached transcriptome-wide significance in the TWAS of CD8^+^ T-cells (Table S5). These transcripts are composed of 56 protein-coding, 24 processed transcripts, five pseudogenes, three nonsense-mediated decay, and two IG genes (Fig. 1C). In this cell population, a protein-coding transcript for the gene, *HEATR2*, was the most significantly associated transcript (*p*-value = 6.46 × 10^−12^). We found that the significant protein-coding transcripts were enriched for 18 pathways, which group into three clusters related to protein secretion, signal transduction, and neuronal processes (Table S8).

#### CD4^±^ T-cells

In CD4^+^ T-cells, 36 transcripts from 35 genes passed transcriptome-wide significance (Table S6). These significant transcripts included 21 protein-coding, 11 processed transcripts, three pseudogenes, and one IG gene (Fig. 1C). *IGKV1D-13*, was the top transcript (*p*-value = 2.94 × 10^−4^). Top protein-coding transcripts from the CD4^+^ T-cell TWAS were enriched for 19 pathways, which segregate into three clusters related to neuronal development, signal transduction, and cell receptor signaling (Table S9).

#### B-cells

The TWAS in B-cells yielded 891 transcripts representing 789 genes that were transcriptome-wide significant (Table S7). The altered transcripts are comprised of 534 protein coding, 242 processed transcripts, 56 nonsense-mediated decay, 46 pseudogenes, 12 IG genes, and one T-cell receptor (TR) gene (Fig. 1C). The most significant (*p*-value = 1.76 × 10^−120^) is the only protein-coding transcript for the gene *FMOD*. Pathway analyses of the protein-coding findings show enrichment for 98 pathways. Pathway clustering results in ten clusters including those related to B-cell activation, apoptotic pathways, cellular starvation, cellular metabolism, nucleic acid metabolism, neuron cell morphology, organ development, glucose metabolism, and cell adhesion and cytoskeleton organization (Table S10).

Overall, individual cell types have unique profiles of transcripts that are differentially expressed between cases and controls (Fig. 1B). However, an overlap of 20 significant transcripts is seen among CD4^+^ T-cells, CD8^+^ T-cells and B-cells, which all are lymphocytes. These overlapping differently expressed transcripts could reflect common functions associated to PPD that are shared by the different lymphocytes cell types. Among the overlapping transcripts are three pathway clusters that involve nervous system development, regulation of signal transduction, and regulation of macromolecule metabolism (Table S11).

### Top Loci in Whole Blood Overlap Findings From Previous Transcriptome Studies of PPD

Three other transcriptome studies of PPD have been performed [22–24]. These previous studies were performed using whole blood and are independent of the current study, which allowed us to test whether our top bulk results overlap the most significant genes in the previous studies.

As shown in Table 1, we found the top 5% of our whole blood results shared genes with the significant genes reported by Landsman et al. Further, for the remaining two studies (Pan et al. and Mehta et al.) we observed overlap between the top 5% of our whole blood results and the top 5% of the reported genes. We used sign tests to compare the overall patterns of results between our results and the previous PPD TWAS. Under the null, the expectation is that 50% of the signs of the overlapping genes will be the same between two independent sets of results. The significance of the observed proportion was evaluated using the binomial distribution. From this, we find that our results were concordant with results by Mehta et al. (*p* < 0.001), which may be due to the small sample sizes of the other studies.

**Table 1:** Overlap of top TWAS findings versus existing transcriptome studies of PPD.

Authors	Year	PMID	Cases	Controls	Tissue	Overlapping genes	*n* Shared direction	sign test *p*-value
Landsman et al.	2017	27816578	6	10	PBMC	10	7	0.17
Pan et al.	2018	29973662	8	10	PBMC	19	7	0.92
Mehta et al.	2021	33664235	15	122	Whole Blood	2894	1793	<0.001

### SNPs and DNA methylation, but not hormones, may regulate PPD-associated transcripts

Whole blood and cell type-specific TWAS identified multiple transcripts that differed between cases and controls. We performed regulation analyses to study whether these differences were regulated by pregnancy-related hormones (estradiol, E2; progesterone, P4; oxytocin; BDNF), DNA methylation, or SNPs measured in the same samples. These analyses followed the model depicted in Fig. S4, which assumes the tested marker regulates transcription (a mediator), which in turn alters PPD risk. As there may be additional mechanisms through which the marker may affect PPD, the model also allows for a direct effect of the marker on PPD. The null hypothesis states that the marker does not regulate differentially expressed transcripts (H_0_: *a* × *b* = 0). SNPs and CpGs were tested as putative regulators if they had a nominal association with case status (*p* < 0.05) and were within a 10 kb window (cis-acting elements) of the genes tested. Hormones were tested as putative regulators if they had a nominal association with case status (*p* < 0.05).

The postpartum period is characterized by a large fluctuation in hormones, making dysregulation as a result of hormone changes an attractive mechanism. We observed significant association between PPD case status and oxytocin levels (*p*-value = 2.38 × 10^−4^), but not for any other measured hormones (Table S1). However, when we examine oxytocin as a potential regulator of transcriptional differences, we did not observe any significant effects (Table S12). This is not to say hormones are not contributing to case-control differences, but we do not see supporting evidence that hormones regulate the specific transcriptional differences observed in this study.

Among the disease-associated DNA methylation sites (10 CpGs in bulk, 158 CpGs in CD8^+^ T-cells, 67 CpGs in CD4^+^ T-cells, 1,520 CpGs in B-cells, 13 CpGs in monocytes, 5 CpGs in granulocytes), we identified five CpGs that were significantly associated with four differentially expressed transcripts in B-cells (*q*-value < 0.1; Table S13). Thus, these methylation marks may serve as potential cis-acting regulators for their corresponding protein-coding transcripts for *CD22*, *CXXC5*, *MYO1D*, and *KCNG1* [31]. It should be noted that methylation was assayed with a commonly used array-based platform resulting in 373,635 high-quality methylation markers, which corresponds to ~1.3% out of the 28.3 million CpGs in the human genome [32]. Thus, it is possible, and likely, that many additional regulatory methylation marks are present that were not assayed in our dataset.

Using our SNP data, we examined whether disease-associated eQTLs (deQTL) exist for any of the differentially expressed transcripts. We tested 36 SNPs in whole blood, 1696 SNPs in CD8^+^ T-cells, 322 SNPs in CD4^+^ T-cells, 15,154 SNPs in B-cells, 149 SNPs in monocytes, and 58 SNPs in granulocytes as putative regulators. No deQTLs were identified for whole blood and granulocytes. However, we detected 17 deQTLs for seven transcripts in CD8^+^ T-cells, seven deQTLs for three transcripts in CD4^+^ T-cells, 523 deQTLs for 124 transcripts in B-cells, and four deQTLs for two transcripts in monocytes (*q*-value < 0.1; Table S14). The majority of the transcripts with deQTLs were protein-coding (78%). These deQTLs are genomic regulators of transcriptional differences associated with PPD and have translational value as potential targets for correcting aberrant transcription.

### PPD deQTLs are enriched for multiple brain eQTLs but not for MDD GWAS loci

We tested whether the PPD deQTLs we identified were enriched for significant MDD GWAS loci [33, 34]. However, we did not observe any overlap between PPD deQTLs and significant MDD GWAS loci. The lack of overlap may be due to differences in ancestry between studies. Our PPD cohort is comprised mainly of Black and Hispanic women, whereas the MDD GWAS was limited to individuals of European ancestry. Alternatively, our results could support literature that PPD is a distinct disorder with a different underlying etiology [35–38].

Further, to examine the potential impact of PPD deQTLs across tissues, we tested whether they overlapped eQTLs in bulk brain tissue or neurons from various brain regions as reported by the most recent GTEx analyses [18, 19]. We found significant enrichment with our deQTLs for eQTLs in nearly all brain regions (Table 2; 10 out of 12, 83.3%), with the exception of amygdala and substantia nigra. However, we did not identify any enrichment for eQTLs in neurons. Although additional investigations would be required, this could suggest that the PPD deQTLs detected in this study do not exert their effects on neurons, but rather non-neuronal cell types, such as glia. In total, there were 45 deQTL containing genes overlapping with eQTLs in various brain tissues (Table S14).

**Table 2:** Overlap of PPD deQTLs in bulk brain tissue or neurons from various brain regions.

	Enrichment Target	Enrichment OR	*p*-value
Bulk eQTLs	Amygdala	2.31	0.06
	Anterior Cingulate Cortex (BA24)	2.32	0.04
	Caudate Basal Ganglia	2.77	0.01
	Cerebellar Hemisphere	2.59	3.02E-03
	Cerebellum	2.58	2.19E-03
	Cortex	2.95	1.05E-03
	Frontal Cortex (BA9)	3.27	9.40E-04
	Hippocampus	2.54	0.03
	Hypothalamus	3.90	5.90E-04
	Nucleus Accumbens (Basal Ganglia)	2.88	2.43E-03
	Putamen (Basal Ganglia)	2.33	0.02
	Substantia Nigra	2.19	0.07
Neuronal eQTLs	Amygdala	1.47	0.39
	Anterior Cingulate Cortex (BA24)	0.73	0.74
	Caudate Basal Ganglia	0.00	1.00
	Cerebellar Hemisphere	0.76	0.72
	Cerebellum	0.00	1.00
	Cortex	1.55	0.37
	Frontal Cortex (BA9)	1.53	0.37
	Hippocampus	2.21	0.16
	Hypothalamus	1.52	0.38
	Nucleus Accumbens (Basal Ganglia)	1.53	0.37
	Putamen (Basal Ganglia)	0.00	1.00

### Convergent evidence implicates pathways most affected in PPD

**Fig. 2:**
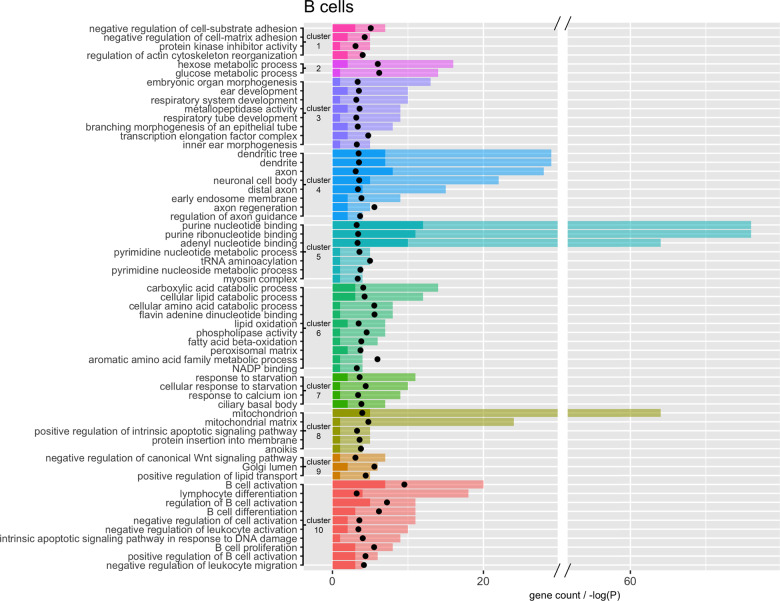
Clusters of significant TWAS pathways in B-cells that contain at least one deQTL gene. Each cluster is represented by a different color. Opaque bars are the total number of genes overlapping the pathway. Solid bars are the number of genes with a deQTL. Black points are -log(p-value) for the pathway enrichment.

Pathway analyses of TWAS results identified pathways that are altered in PPD cases compared with controls. However, RNA transcription is potentially regulated by many biological processes. Our analyses suggested that SNPs (deQTLs), and to a lesser extent DNA methylation, may be involved in the regulation of PPD-related transcriptional differences. By examining the overlap of these pathways with deQTLs we can identify which pathways are initially disrupted by genetic loci in cases compared to the effect of other factors. We identified 138 transcripts regulated by deQTLs in whole blood and across the five cell types examined. More specifically, 124 of these transcripts were found in B-cells and overlap pathways in every pathway cluster identified. Figure 2 illustrates the B-cell pathways that contain at least one deQTL containing gene organized by cluster. Additional results for other cell types can be found in Supplemental Tables. Overall, our results suggest the pathway clusters shown in Fig. 2 may be potentially dysregulated due to genetic factors in women with PPD.

## Discussion

To generate novel hypotheses for PPD disease pathology, we studied the biological underpinnings of PPD in a large cohort of women six weeks after childbirth. Results showed cell type-specific transcriptional differences associated with PPD, with a majority of the changes seen in B-cells. Furthermore, these associations were significantly overrepresented in multiple sets of pathways. These pathways reflected the significant effects of SNPs regulating the PPD-associated transcriptional changes. This constitutes a convergence of evidence with data from two different biological mechanisms.

Pregnancy is characterized by substantial changes in multiple physiological systems. Failure to return to pre-pregnancy levels during the postpartum period may contribute to PPD symptoms, making these systems candidate mechanisms for PPD. Our association and pathway results implicate two, potentially co-occurring, such mechanisms: B-cell activation and insulin resistance (IR). In addition to showing pregnancy-related changes, both mechanisms have previously been linked to depression [39–44].

Our results specifically implicate B-cell activation (Fig. 2, cluster 10), which plays a critical role in the immune system. B-cells becomes activated when their receptor recognizes an antigen and binds to it. Activated B-cells then produce antibodies, along with secreting pro- and anti-inflammatory factors. During pregnancy, B-cells undergo dynamic changes as the maternal immune system has to balance tolerance of the foreign‐growing fetus with maintaining vigilance against pathogens [45, 46]. Thus, B-cell concentrations are significantly lower during the third trimester and immediately following delivery compared to non-pregnant women, but levels typically return to those seen in non-pregnant women by six-weeks postpartum [47].

A growing body of evidence suggests that inflammatory processes may play a significant role in PPD [24, 48–50]. However, the specific role of B-cells has yet to be elucidated. Recent work has shown an increase in B-cell densities in the brains of those with mood disorders compared to controls [39]. Furthermore, in whole blood, altered B-cell homeostasis was observed in those with MDD compared to controls [40, 41]. A possible mechanism contributing to increased B-cell activation could be related to autoimmunity [51]. Depression is often co-morbid with autoimmune disease; risk of depression is 1.25–3.56 times higher in people with autoimmune disease than without [52–54]. Additionally, a feature of many autoimmune disorders is a loss of B-cell tolerance coinciding with the inappropriate production of autoantibodies [51, 55]. Thus, an aberrant autoimmune response could potentially contribute to PPD.

Further, we did not observe significant differences in B-cell proportions between cases and controls (*p*-value = 0.78). As multiple subtypes of B-cells exist, it may be that we did not observe differences in overall B-cell proportions but there may be differences in more specific B-cell subsets.

Not only do we observe a pathway cluster, composed of 12 pathways, directly related to B-cell activation (cluster 10), we see multiple pathway clusters associated with cellular metabolism, which supports our hypothesis of B-cell activation. Activation initializes cellular reprogramming of quiescent naïve B-cells to drive re-entry into the cell cycle [56]. This rapid expansion requires the production of biomolecules (lipids, proteins, nucleotides in clusters 2, 5, 6, and 9) at an increased rate. Additionally, work in mice has shown that B-cell activation results in increased glucose uptake (cluster 2) and mitochondrial remodeling (cluster 8) [56]. Upon B-cell activation, not only is there a slew of metabolic changes, but there are changes to the cellular structure (cluster 1). Antigen binding triggers substantial remodeling of the cell cytoskeleton, which induces cell spreading, the formation of the immune synapse, and the gathering of antigen for endocytosis [57]. Additionally, apoptosis is a carefully regulated process through the lifecycle of B-cells. Disruptions to apoptotic pathways (cluster 8) affect multiple processes including homeostasis, quality control of the antibody response, and tolerance [58].

The second implicated mechanism, insulin resistance (IR), is supported by several factors. Insulin promotes the absorption of excess blood glucose into other tissues for energy storage. IR occurs when cells become insensitive to the effects of insulin leading to a buildup of blood glucose and insulin. Starting in the second trimester of pregnancy, insulin sensitivity is progressively reduced as much as 60–80% [59]. This coincides with steady increases in insulin [60]. These changes serve as a physiological adaptation of the mother to ensure adequate carbohydrate supply for the rapidly growing fetus [61]. After delivery, insulin returns to pre-pregnancy levels [62–64].

IR is a risk factor for depression. Rodent studies have shown that brain IR alters dopamine turnover and induces anxiety and depressive-like behaviors in mice [65]. In humans, greater glycemic variability has been associated with negative moods [66]. IR typically predates the development of diabetes. A meta-analysis of 27 studies demonstrated that depression is significantly associated with hyperglycemia for both type 1 and type 2 diabetes [67]. Studies further suggest that insulin-sensitizing agents could play a significant role in the treatment of major depression, particularly in patients with documented IR [68, 69]. Pregnancy is known to increase the risk of developing Type 2 diabetes after giving birth [70]. Furthermore, pre-pregnancy or gestational diabetes was independently associated with perinatal depression, including new onset of PPD [71–73].

We tested whether genes implicated by our top results were significantly overrepresented for genes related to A1C [74] and IR [75] in whole blood. Hemoglobin A1C levels are measure of a person’s blood sugar levels over the past three months and are highly correlated with measures of IR [76]. We found the top 5% of our whole blood findings for PPD were enriched for the top 5% of associations with A1C (*p*-value = 4.79 × 10^−7^) and IR (*p*-values = 0.04). Databases can not directly implicate IR as such a pathway does not exist. IR is a disorder characterized by disruptions of multiple biological functions. However, IR can be implicated by nearly all clusters in our pathway analyses (Fig. 2). With the evidence linking IR and B-cells [77, 78], it is reasonable to observe a signature of IR in B-cells. For example, B-cells contribute to the development of IR (cluster 10). These cells can promote IR through T-cell modulation and production of pathogenic antibodies [77, 78]. Insulin signaling regulates diverse cellular functions including metabolic pathways, apoptosis, mitogenesis, and membrane trafficking through protein kinases (cluster 1) [79, 80]. Insulin directly affects glucose metabolic processes (cluster 2). Circulating levels of purines (cluster 5) [81, 82], amino acids, and fatty acids (cluster 6) [83, 84] are also associated with IR. The administration of carboxylic acids (cluster 6) improved glycemic control, potentially by reducing IR [85]. IR may lead to inadequate intracellular glucose potentially leading to apoptosis and intracellular starvation (cluster 7 and 8) [86]. Wnt signaling (cluster 9) is involved in the regulation of glucose homeostasis in multiple organs, particularly in insulin-responsive tissues [87].

A number of limitations of the present study should be mentioned. While we studied blood, the pathogenic processes for PPD most likely manifest in the brain. It is likely that in studying blood, other possible PPD-related mechanisms might have been missed. However, there is cross-talk between the two tissues across the blood-brain barrier [88]. This would allow altered B-cell activation and IR to affect the brain and be observed in our study. Furthermore, we observed deQTLs that affect genes in both blood and brain, specifically in brain regions implicated in mood disorders (e.g., hippocampus, cingulate cortex, frontal cortex). These deQTLs can be studied in model systems for functional follow-up to evaluate causality and their downstream biological effects [89]. Additionally, the B-cell activation and IR hypotheses for PPD requires further validation through direct measurements in PPD cases versus controls.

In conclusion, we have performed the largest and most comprehensive biological interrogation of PPD, to date. Our results suggest that PPD is associated with an increase in B-cell activation, a finding previously unreported in the literature. While we do not know the precise mechanisms behind this increase in B-cell activation, we hypothesize it could be due to co-occurring dysregulation in IR. Additionally, we identified genetic variants, deQTLs, that regulate, in part, the transcriptional differences between cases and controls. Our findings require further validation and follow-up studies. However, these novel hypotheses for PPD provide promising avenues for future research.

## Methods

### Study population

Detailed information about the study can be found elsewhere [90]. Briefly, we followed the 2010 US Census terminology for describing the self-reported “race” and “ethnicity” (Hispanic or Non-Hispanic) of subjects. We refer to the participants as Asian, Latina (“of Latino, Hispanic, or Spanish origin”), Black (or African-American), and White (i.e., European ancestry, non-Hispanic).

Recruitment of postpartum women aged 17–45 years occurred from 9/2012 to 6/2016 in four outpatient obstetrical clinics (University of North Carolina Women’s Hospital, Wake County Health Department, Alamance County Health Department, East Carolina University School of Medicine) during routine six-week postpartum visits (± 1–2 weeks). Detailed recruitment procedures can be found in Supplemental Methods.

Case-control status was determined using clinical interview. All women attending these clinics were first screened for study inclusion using the Edinburgh Postnatal Depression Scale (EPDS). The 10-item EPDS is a commonly used PPD screening instrument. High EPDS scores are consistent with a PPD diagnosis by structured clinical interview [91]. Women with high EPDS scores (≥11) or low EPDS scores (≤7) were invited to participate. All women then had PPD case status determined using the MINI diagnostic interview. For a full list of inclusion/exclusion criteria, see Supplemental Methods. Briefly, all participants included no indication of MDD during the first or second trimesters of pregnancy, singleton pregnancy, and live term birth (≥34 weeks gestation). This study was approved by the University of North Carolina Institutional Review Board Committee for the Protection of Human Subjects. All subjects provided written informed consent and signed the Health Insurance Portability and Accountability Act release.

### Subject Assessments

All participants were administered the MINI International Neuropsychiatric Interview (MINI-Plus, version 6.0), a structured clinical interview for the assessment of psychiatric disorders [92, 93]. Experienced and certified (κ > 0.8 versus criterion ratings) psychiatric research nurses working in each clinic setting administered the MINI-Plus. Cases for this study were defined by having current MDD as assessed by the MINI-Plus. Controls did not have current MDD using the MINI-Plus. All study procedures could be performed in Spanish with a native speaker.

### Biological Sampling

Peripheral blood was sampled and immediately processed on-site at the time of subject assessment. For plasma separation, blood aliquots were centrifuged at 3300 rpm for 10 min at room temperature immediately after sampling. For serum separation, blood aliquots were centrifuged at 3300 rpm for 10 min at 2–8 °C, 60 min after blood draw. All plasma and serum samples were then snap-frozen and kept at −80 °C until analysis. Aliquots were drawn into PAXgene RNA tubes and stored at −80 °C until RNA extraction. Genomic DNA was extracted from aliquots of whole blood using Qiagen Autopure LS, which utilized Qiagen Puregene chemistry. Samples that were missing, had insufficient sample, or that did not meet minimum detection thresholds were excluded from analyses.

### RNA Sequencing

For RNA extraction, samples are pulled from −80 °C freezers, allowed to thaw at +4 °C overnight, and extracted using the QIAsymphony platform. A detailed RNA extraction protocol can be found in Supplemental Methods. Fresh-frozen total RNA was prepared for sequencing following the Nugen Ovation Human Blood RNA-seq library prep kit according to the manufacturer’s instructions. RNA libraries were sequenced as 2 × 50 bp paired-end reads with 24 samples per lane on an Illumina HiSeq 4000 sequencer. Each sample was run on two different lanes at two different times. Samples were balanced by case status, age, race, ethnicity, and recruitment site across sequencing pools to reduce technical biases. Preliminary sample and read quality control (QC) was performed using FastQC using default settings. Briefly, raw sequence reads are read in and reports are generated on read quality and composition. No samples were dropped or required resequencing. Reads were aligned with HISAT2 (v2.1.0) and transcriptomes were reconstructed using StringTie (v.1.3.3), both within the rnacocktail pipeline [94]. Reads from runs 1 and 2 for every sample were merged prior to quantification. Reference transcriptome was downloaded from ENSEMBL (GRCh37, release 92; http://ftp.ensembl.org/pub/grch37/release-92/gtf/homo_sapiens/Homo_sapiens.GRCh37.87.gtf.gz) [26] and used for alignment, transcriptome reconstruction, and quantification steps. This reference includes all available biotypes including protein coding genes, as well as pseudogenes, lncRNA, and ncRNA. Following transcriptome assembly, the StringTie merge option was used to combine all assembled transcriptomes across all samples and then re-quantified against the merged transcriptome (stringtie -eB) so expression measures are consistent across all samples. Transcripts were excluded if they were depletion targets for library prep (ENSEMBL gene_biotype “rRNA”, *HBA1*, *HBA2*, *HBB*, *HBD*), unannotated (not associated with an ENSEMBL ID), present in < 1% of samples, had an average TPM < 1 (low expression outlier) or > 20,000 (high expression outlier). Following this quality control, data for 108,474 transcripts remained for association testing.

For association testing, technical variables were measured for each sample including: i) the total number of reads, the number of uniquely aligned reads, and the proportion of reads aligned using StringTie, ii) sequencing pool, and iii) calculation of the first ten principal components across all transcript counts for depletion targets for library prep (ENSEMBL gene_biotype “rRNA”, *HBA1*, *HBA2*, *HBB*, *HBD*). Final association models included maternal age, race/ethnicity, estimated cell proportions, proportion of reads aligned, number of uniquely aligned reads, and sequencing pool. Additionally, principal components of TPM values were used to capture any remaining unmeasured source of variation. One principal component (PC1) was included based on the scree test. Lastly, multidimensional outliers were excluded, resulting in data for 482 cases and 859 controls. Quantile-quantile plots for each cell type–specific TWAS (Fig. 1A), along with TWAS of permuted case-control status for each analysis yielded average lambdas of approximately 1 (Figure S1), indicated no evidence of test-statistic inflation under the empirical null.

### DNA Methylation Assessment

A detailed DNA methylation pipeline can be found in Supplemental Methods. DNA sample bisulfite conversion and microarray hybridization were through the Illumina Fast Track Genotyping service. DNA methylation was assessed using the Infinium Human 450k array. Quality control steps are described elsewhere [95]. Briefly, we employed a stringent quality control pipeline comprised of the following steps: i) removal of samples with > 1% of probes with detection P-value > 0.001, ii) removal of probes with > 1% of samples with detection P-value > 0.001, iii) removal of cross-hybridizing probes, iv) and probes containing a SNP with minor allele frequency > 1% within 10 bp of the single-base extension position [96]. Normalization of the DNA methylation data was performed using the BMIQ function [97] within the minfi package. Following quality control of probes, data for 373,635 CpGs remained for regulation analyses.

Residuals were used for regulation (deQTL) analyses. Covariates were selected using multiple regression analyses in RaMWAS [98] from a pool of multiple types of variables (see Supplemental Methods for description of variables tested). Final association models included maternal age, race/ethnicity, estimated cell proportions, slide and array (batch), and median methylated and unmethylated signal intensities, and three PCs from raw control probes (PCs 2, 8, 10). Additionally, PCs of beta values were used to capture any remaining unmeasured source of variation. PCs 1–5 were included based on the scree test. As a final step, multidimensional outliers across PC1–15 were identified using the mvoutliers R package and excluded, resulting in data for 503 cases and 897 controls.

### SNP Genotyping

Genotypes were assessed using the Illumina Multi-Ethnic Genome Arrays (MEGA; Illumina, San Diego, CA, USA) through the Illumina Fast Track Genotyping service. GenomeStudio software version 2.0 (Illumina, San Diego, CA, USA) was used to call genotypes from raw Illumina data. We have described our quality control procedures for SNPs elsewhere [99, 100]. Briefly, SNPs are removed for bad genome mapping of array probe, missingness (>0.01), and low MAF (<0.01). Any individual with high missingness was excluded (>0.01). Genotypes were imputed against the Haplotype Reference Consortium [101] using the University of Michigan Imputation Server [102]. Following imputation, genotypes underwent another round of quality control. Genotypes were excluded for having low quality scores (*r*^2^ < 0.8), missingness (>0.01), and low MAF (<0.01). Again, any individual with high missingness was excluded (>0.01).

For deQTL analyses, principal components of SNPs were used to capture any unmeasured source of variation in our genetic data (e.g population stratification). Two principal components (PCs 1 and 2) were included based on the scree test, resulting in data for 487 cases and 864 controls.

### Cell type-specific analyses

Whole blood contains a mixture of different cell types. Cell type-specific TWAS are critical as expression changes may remain undetectable in whole blood as changes may cancel each other out when they have opposites affects across cell types or involve effects of low abundant cells that are diluted by effects of common cell types [12, 18]. Cell type-specific TWAS also improve the biological interpretation of findings as knowing the cell type in which the change occurred may provide further clues about the underlying biological mechanisms. Therefore, we performed cell type-specific deconvolution analyses, which were first introduced about 20 years ago [103]. Most of the initial deconvolution papers include sections showing the validity of the approach. For example, the 2010 paper by Shen-Orr [29] experimentally validated the method through tests with predesigned mixtures.

For TWAS, cell type-specific analyses were conducted using a deconvolution approach described and validated previously [16, 28, 29]. Briefly, cell proportions are estimated from bulk (cellularly heterogenous) DNA methylation data using available reference panels [27]. These predicted cell type proportions are then used to test case-control differences on a cell type-specific level using all study samples with available bulk transcription data. The statistical model we use is:

$$Y^{bulk} = \mathop {\sum }\limits_{c = 1}^{n_c} m_cP_c + \mathop {\sum }\limits_{c = 1}^{n_c} m_c^{PPD}\left( {PPD \times P_c} \right) + E$$

Thus, measurements from bulk tissue *Y*^*bulk*^ are regressed on *c* = 1 to *n*_*c*_, cell type proportions *P*_*c*_, and the product of disease status for PPD coded as 0 or 1 by cell type proportions (*PPD* × *P*_*c*_). The model allows for covariates (not shown) and residual effects *E*. Coefficient *m*_*c*_ is the effect of cell type *c*. The case-control difference $m_c^{PPD}$ for cell type *c* is used to test the null hypothesis that cell type means are equal for cases and controls. Note that the model has no constant due to $\mathop {\sum }\nolimits_{c = 1}^{n_c} P_c \cong 1$. Alternatively, the model is sometimes written with a constant whereby one of the cell type proportions is omitted [17] but this produces identical results [16, 28, 29].

### Pathway analyses

Pathway analyses were performed with Reactome [104] and GO [105, 106] databases. These analyses also used circular permutations (For a detailed description see Circular Permutations section of Supplemental Methods) that properly control the Type I error in the presence of correlated sites. Furthermore, as the permutations are performed on a marker level they also properly account for gene size, as larger genes with more markers are more likely to be among the top results in the permutations. Specifically, we first mapped each marker to genes (Ensembl gene annotations GRCh37, release 92: ftp://ftp.ensembl.org/pub/grch37/release-92/) [26] using the Bioconductor GRanges package. Gene boundaries were extended to include a 10 kb upstream flank (i.e., promoters). Markers were allowed to map to multiple genes if their genomic position overlapped multiple unique gene annotations. After mapping, we performed 10,000 circular permutations at the marker level. For each permutation, a two-by-two table was created by cross classifying whether or not the genes were among the top findings for each analysis (TWAS) versus whether or not the gene was in the tested pathway. Each gene was counted only once when creating this table (thus, if there were three markers in the gene, this was counted as one and not as three). Cramér’s V (sometimes referred to as Cramér’s phi) was used as the test statistic to measure whether genes from the pathway were overrepresented among the top analysis results. P values were calculated as the proportion of permutations that yielded a value equal to or greater than that of Cramér’s V observed in the empirical data. A minimum of three input genes were required to be present in each queried pathway and were considered significant after controlling the family-wide error rate at *α* = 0.05. Pathway tests were run on all markers with a *q*-value < 0.1. As many pathways share a large number of common gene members, we used the Louvain method [107] in the igraph R package to cluster enriched pathways by similarity.

### Regulation Analyses

To study regulatory effects of SNPs, we perform mediation analyses using the model in Fig. S4 (covariates not shown) assuming that the SNP regulates (path *a*) transcription (a mediator), which in turn alters (path *b*) PPD risk. As there may be additional mechanisms through which the marker may affect PPD, the model also allows for a direct effect of the SNP (path *c*’) on PPD. The null hypothesis states that the marker does not regulate differentially expressed transcripts (H_0_: *a* × *b* = 0).

The model in Fig. S4 is the standard approach underlying Mendelian Randomization [108] that aims to improve causal inferences in correlational studies. In this model, the SNP is the instrumental variable where the direction of effect has to be from SNP to transcript abundance (path a) and SNP to PPD status (path c’). Thus, the causal direction cannot be reversed as neither gene expression nor PPD status can change the SNP. The direction of effect from transcript abundance to PPD status (path b) can in principle be reversed. However, if we reverse the direction of effect there can no longer be an indirect effect of the SNP on PPD so that in these instances the null hypothesis, H0: *a* × *b* = 0, is true. Therefore, rejecting the null hypothesis in the SNP regulatory analyses essentially provides evidence for the causal direction of effects displayed in Fig. S4 where transcriptional changes alter PPD risk and not the other way around.

We also used the model depicted in Fig. S4 to study possible regulatory effects of hormones and DNA methylation. However, as both the direction of path a and path c’ can be reversed it no longer provides evidence for the causal direction of effects so strictly speaking significant findings cannot be interpreted as meaning that hormones and DNA methylation are regulators.

For each gene with a differentially expressed transcript, all annotated transcripts with expression data were tested. SNPs and CpGs were tested as putative regulators if they had a nominal association with case status (*p* < 0.05) in a 10 kb window of the genes tested. Hormones were tested as putative regulators if they had a nominal association with case status (*p* < 0.05). This pre-selection avoids running regulatory analyses with a large number of markers (SNPs, CpGs, hormones), the majority of which cannot be regulators because they do not affect transcript abundance. All raw data had their respective covariates regressed out prior to mediation testing. Causal mediation analyses were conducted with the mediate package (v4.5.0) in R. Specifically, we used the mediate function which implements a quasi-Bayesian approach with 1000 to 1,000,000 Monte Carlo draws for the approximation of the *p*-values for the mediation effect [109]. All analyses begin with 1000 simulations. If a p-value cannot be approximated (*p*-value = 0), another round is performed using 10,000 simulations. This process continutes with a 10-fold increment in simulations until a p-value can be approximated, or the number of simulations reaches 1,000,000. A *q*-value < 0.1 was used to declare significance [30].

## Supplementary information

Supplemental Material

Figure S1

Figure S2

Figure S3

Figure S4

Supplemental Tables

## Data Availability

Full transcriptomic, DNA methylation, and SNP data used to support the findings of this study have, or will be, deposited in dbGaP (accession: phs002103.v1.p1). [https://www.ncbi.nlm.nih.gov/projects/gap/cgi-bin/study.cgi?study_id=phs002103.v1.p1]. but will be embargoed until results from full datasets are in press.
