# Supplementary material for: Transcriptome-wide association study for postpartum depression implicates altered B-cell activation and insulin resistance
Source: Mol Psychiatry. 2022 Apr 1;27(6):2858–67. doi: 10.1038/s41380-022-01525-7 (PMC9156403; doi:10.1038/s41380-022-01525-7)
Supplement: Supplementary file 1 — Supplemental Material [file 41380_2022_1525_MOESM1_ESM.docx]

# SUPPLEMENTAL MATERIAL

## **SUPPLEMENTAL METHODS**

Study Population

Detailed information about the study can be found elsewhere ^1^. Briefly, we followed the 2010 US Census terminology for describing the self-reported “race” and “ethnicity” (Hispanic or Non-Hispanic) of subjects. We refer to the participants as Asian, Latina (“of Latino, Hispanic, or Spanish origin”), Black (or African-American), and White (i.e., European ancestry, non-Hispanic).

Recruitment of postpartum women aged 17-45 years occurred from 9/2012 to 6/2016 in four outpatient obstetrical clinics (University of North Carolina Women’s Hospital, Wake County Health Department, Alamance County Health Department, East Carolina University School of Medicine) during routine six-week postpartum visits (± 1-2 weeks). As non-European ancestry was a key interest, we took multiple steps to enhance recruitment of Black and Latina women, including recruitment advertisements in free and Spanish language newspapers, local internet advertisements, employment of research coordinators to recruit in clinics (including Spanish speaking coordinators), access to translators, and full protocol in Spanish.

Case-control status was determined using clinical interview. All women attending these clinics were first screened for study inclusion using the Edinburgh Postnatal Depression Scale (EPDS). The 10-item EPDS is a commonly used PPD screening instrument. High EPDS scores are consistent with a PPD diagnosis by structured clinical interview^2^. Women with high EPDS scores (≥11) or low EPDS scores (≤7) were invited to participate. All women then had PPD case status determined using the MINI diagnostic interview. Inclusion criteria for all participants included no indication of MDD during the first or second trimesters of pregnancy, singleton pregnancy, live term birth (≥ 34 weeks gestation), and hematocrit ≥ 30% after delivery (so that blood sampling in this protocol was clinically reasonable). Exclusion criteria for all participants included lifetime mood disorder other than MDD (e.g., bipolar disorder), lifetime psychotic disorder, lifetime alcohol or illicit substance dependence, current significant alcohol or illicit drug use, current primary anxiety disorder (e.g., post-traumatic stress disorder, obsessive compulsive disorder), major medical illness (e.g., type-2 diabetes), any serious adverse birth outcome, clinically significant anemia, and evidence of untreated significant thyroid disease or infection. This study was approved by the University of North Carolina Institutional Review Board Committee for the Protection of Human Subjects. All subjects provided written informed consent and signed the Health Insurance Portability and Accountability Act release.

Subject Assessments

All participants were administered the MINI International Neuropsychiatric Interview (MINI-Plus, version 6.0), a structured clinical interview for the assessment of psychiatric disorders ^3,4^. Experienced and certified (κ > 0.8 versus criterion ratings) psychiatric research nurses working in each clinic setting administered the MINI-Plus. Cases for this study were defined by having current MDD as assessed by the MINI-Plus. Controls did not have current MDD using the MINI-Plus. All study procedures could be performed in Spanish with a native speaker.

Study coordinators used a structured form to abstract additional data from the medical record: EPDS at visit; previous psychiatric diagnoses; pregnancy/birth events and complications; birth outcomes; and routine clinical laboratory testing results (e.g., hematocrit and urinary drug screen at birth). Research coordinators measured height via stadiometer and weight. Using a structured interview, socioeconomic status, breastfeeding behaviors, and menstrual status were assessed.

RNA Sequencing

For RNA extraction, samples are pulled from -80^o^C freezers and allowed to thaw at +4^o^C overnight.  Samples are acclimated to room temperature for a minimum of two hours before initiating RNA extraction processing.  Samples are mixed by inversion ten (10) times prior to centrifugation.  PAXgene RNA tubes are centrifuged at 3000x g for ten (10) minutes.  Supernatants are poured off and tubes are inverted to dry the cell pellets.  Two hundred and eighty microliters (280ul) of reagent QSX2 and twenty microliters (20ul) of Proteinase K are added to each tube, then vortexed in order to resuspend cell pellets.  Two hundred microliters (200ul) of binding buffer BR2 is added prior to initiating extraction on the QIAsymphony.  Samples are loaded onto the deck of the QIAsymphony.  The PAXRNA_CR22240_ID2776 protocol is run.  Upon completion of the QIAsymphony protocol the RNA eluates are heated at 80^o^C for ten (10) minutes (denaturing the RNA) in the Qiagen Elution Microtube CL heating/cooling adapter.  Samples are cooled on wet ice (+4^o^C) for at least ten (10) minutes.  RNA eluates are transferred from Elution Microtubes CL rack into Micronic 0.75mL tubes where a unique 2D barcode is assigned to each stock sample.

Fresh-frozen total RNA was prepared for sequencing following the Nugen Ovation Human Blood RNA-seq library prep kit according to the manufacturer’s instructions. RNA libraries were sequenced as 2 × 50 bp paired-end reads with 24 samples per lane on an Illumina HiSeq 4000 sequencer. Each sample was run on two different lanes at two different times. Samples were balanced by case status, age, race, ethnicity, and recruitment site across sequencing pools to reduce technical biases. Preliminary sample and read quality control (QC) was performed using FastQC using default settings. Briefly, raw sequence reads are read in and reports are generated on read quality and composition. No samples were dropped or required resequencing. Reads were aligned with HISAT2 (v2.1.0) and transcriptomes were reconstructed using StringTie (v.1.3.3), both within the rnacocktail pipeline ^5^. Reads from runs 1 and 2 for every sample were merged prior to quantification. Reference transcriptome was downloaded from ENSEMBL (GRCh37, release 92; <http://ftp.ensembl.org/pub/grch37/release-92/gtf/homo_sapiens/Homo_sapiens.GRCh37.87.gtf.gz>) ^6^ and used for alignment, transcriptome reconstruction, and quantification steps. This reference includes all available biotypes including protein coding genes, as well as pseudogenes, lncRNA, and ncRNA. Following transcriptome assembly, the StringTie merge option was used to combine all assembled transcriptomes across all samples and then re-quantified against the merged transcriptome (stringtie -eB) so expression measures are consistent across all samples. Transcripts were excluded if they were depletion targets for library prep (ENSEMBL gene_biotype “rRNA”, *HBA1*, *HBA2*, *HBB*, *HBD*), unannotated (not associated with an ENSEMBL ID), present in < 1% of samples, had an average TPM < 1 (low expression outlier) or > 20,000 (high expression outlier). Following this quality control, data for 108,474 transcripts remained for association testing.

For association testing, technical variables were measured for each sample including: i) the total number of reads, the number of uniquely aligned reads, and the proportion of reads aligned using StringTie, ii) sequencing pool, and iii) calculation of the first ten principal components across all transcript counts for depletion targets for library prep (ENSEMBL gene_biotype “rRNA”, *HBA1*, *HBA2*, *HBB*, *HBD*). Final association models included maternal age, race/ethnicity, estimated cell proportions, proportion of reads aligned, number of uniquely aligned reads, and sequencing pool. Additionally, principal components of TPM values were used to capture any remaining unmeasured source of variation. One principal component (PC1) was included based on the scree test. Lastly, multidimensional outliers were excluded, resulting in data for 482 cases and 859 controls. Quantile-quantile plots for each cell type–specific TWAS (**FIGURE 1A**), along with TWAS of permuted case-control status for each analysis yielded average lambdas of approximately 1 (**Figure S1**), indicated no evidence of test-statistic inflation under the empirical null.

DNA Methylation Assessment

DNA sample bisulfite conversion and microarray hybridization were through the Illumina Fast Track Genotyping service. Genomic DNA underwent bisulfite conversion using the EZ-96 DNA Methylation Kit (Zymo Research, Catalog #: D5004) prior to array hybridization. Converted samples were processed using Infinium chemistry, based on the Infinium LCG Assay Guide. Samples are loaded onto a 12x1 beadchip with InfiniumHumanMethylation450_v1 content. All chips were imaged on an Illumina HiScan or iScan, on unique scanners.

Quality control steps are described elsewhere ^7^. In summary, signal intensities from raw IDAT files were imported in to the R programming environment using the read.metharray.exp function of the minfi package ^8^. We employed a stringent quality control pipeline comprised of the following steps: i) removal of samples with > 1% of probes with detection P-value > 0.001, ii) removal of probes with > 1% of samples with detection P-value > 0.001, iii) removal of cross-hybridizing probes, iv) and probes containing a SNP with minor allele frequency > 1% within 10 bp of the single-base extension position ^9^. Normalization of the DNA methylation data was performed using the BMIQ function ^10^ within the minfi package. Following quality control of probes, data for 373,635 CpGs remained for regulation analyses.

Residuals were used for regulation (deQTL) analyses. Covariates were selected using multiple regression analyses in RaMWAS ^11^ from a pool of multiple typs of variables. Technical variables were generated from the array control probes including: i) median bisulfite conversion percentage for each sample using the bscon() function within the wateRmelon package ^12^, ii) median methylated and unmethylated signal intensities, and iii) calculation of the first ten principal components across all raw control probe intensities. Due to the small proportion of natural killer (NK) cells, they were excluded from covariate selection and the remaining cell types (CD8T, CD4T, Bcell, monocytes, granulocytes) were re-normalized so their sum equals one. This is the standard approach that ensures the estimated proportions sum to one (i.e. 100% of investigated cells) for each participant. Final association models included maternal age, race/ethnicity, estimated cell proportions, slide and array (batch), and median methylated and unmethylated signal intensities, and three PCs from raw control probes (PCs 2, 8, 10). Additionally, principal components of beta values were used to capture any remaining unmeasured source of variation. Five principal components (PC 1-5) were included based on the scree test. As a final step, multidimensional outliers across PC1-15 were identified using the mvoutliers R package and excluded, resulting in data for 503 cases and 897 controls. Smoking scores were calculated for all samples with available DNA methylation data ^13,14^. These scores were also included in post-hoc TWAS analyses but were not included in the final association tests as they resulted in marginal changes in P values and rankings of top tests.

## Circular permutations

For our pathway analyses we used circular permutations ^15^ to account for dependency between markers. To further demonstrate the approach, case-control data were simulated assuming the null hypothesis (i.e., no case control differences) was true for 10,000 sites. The correlation between adjacent sites ranged from 0 to 0.99 and decayed as a function of the distance between the sites (aka, autoregressive model of order 1 or simplex model). For each chosen value of the site correlation, we performed 10,000 simulations and counted the proportion of tests that were rejected at a P-value threshold of α=0.05. Thus, proper control of the Type I error implies that approximately 5% of the tests are rejected. For the sake of comparison, the simulated data was also analyzed using Fisher’s exact test that assumes independent sites. **Figure S3** shows that the use of Fisher’s exact test leads to an excess of false positive findings when sites are correlated. For example, when the correlation between adjacent sites is 0.5, there is a two-fold inflation the number of significant tests and this increases to 16-fold inflation if the correlation is 0.99. The use of circular permutations, however, accurately controls the type I error rate even if correlations between adjacent sites are as high as 0.99.

## **SUPPLEMENTAL RESULTS**

## Correlation of CBC with DNAm Estimates of Cell Proportions

A total of 1,416 individuals had DNA methylation assessed. For a subset of participants (n = 1,271), complete blood counts (CBC) were also measured. This allows us to compare the cell proportions calculated from DNA methylation data with direct cell counts standardly measured in medical clinics. This correlation follow the replication performed by Koestler et al to validate their method of cell proportion data from DNA methylation arrays ^16,17^. Proportions of lymphocytes, monocytes, basophils, eosinophils and neutrophils were assessed in whole-blood by CBC with differential. **Table S16** and **Table S17** show cell type proportions measured by CBC and DNA methylation, respectively. In order to more directly compare CBC cell proportions with those derived from DNA methylation, we focus on three cell types: lymphocytes, monocytes, and granulocytes. For the CBC data, granulocytes are defined as basophils, eosinophils and neutrophils. For the DNA methylation data, lymphocytes are defined as CD8^+^ T-cells, CD4^+^ T-cells, B-cells, and natural killer cells.

As part of the study design, there were 34 samples with at least two (up to three) replicates of DNA methylation arrays. With this, we are able to calculate intra-class correlation coefficients (ICCs) to demonstrate a high-degree of consistency in the predicted cell-type proportions among technical replicates (**Table S17**). We observed ICC values that range from 0.86 – 0.99, illustrating high levels of consistency.

Examining the correlation between the predicted percentage of lymphocytes from DNA methylation and the percentage of lymphocytes via CBC (**Fig. S2a**) demonstrated a high correlation (r = 0.93; p < 2.2 x 10^-16^). Similarly, we also observed significant correlations between monocyte (r = 0.49; p < 2.2 x 10^-16^; **Fig. S2b**) and granulocyte proportions (r = 0.94; p < 2.2 x 10^-16^; **Fig. S2c**).

## Deconvolution Analyses

The cell-type specific deconvolution analyses were first introduced about 20 years ago ^18^. Most of the initial deconvolution papers have sections showing the validity of the approach. For example, the 2010 paper by Shen-Orr ^19^ experimentally validated the method through tests with predesigned mixtures. The method is frequently used to deconvolute with bulk RNA-seq data where deconvolution results are published in high impact journals (e.g., CIBERSORT, one of the many packages that can perform deconvolution, is cites 1,945 times with multiple paper in journals such as *Nature* and *Science*) and has been used by reputable consortia such as GTEx ^20^.

**Figure S5** provides some intuition of how this is achieved using a hypothetical example involving a deconvolution of bulk brain tissue composed of neurons and glial cells.


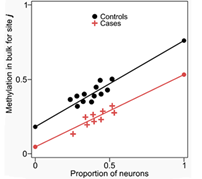


### **Figure S5** Deconvolution of cell-type specific effects from bulk data

Following this example, bulk data and proportions of neurons/glia will differ between subjects, we can regress bulk levels (Y-axis) on the proportion of neurons (X-axis). The slope of the regression line conveys information about the mean expression levels in neurons and glial. For example, extrapolating the regression line to the point where the proportion of neurons is zero (i.e., there are only glia cells) estimates the group mean expression in glia, and extrapolation to the point where the proportion of neurons is one estimates the group mean expression in neurons. By allowing the regression lines to differ between cases and controls, we obtain different predicted cell-type-specific group means that can be tested for significance using standard statistical tests.

More formally, the statistical model we fit is:

$$Y^{bulk}= \sum_{c=1}^{n_{c}} m_{c}P_{c}+ \sum_{c=1}^{n_{c}} m_{c}^{PPD}\left( PPD \times P_{c} \right)+E$$

Thus, measurements from bulk tissue $Y^{bulk}$ are regressed on $c=1$ to $n_{c}$, cell type proportions $P_{c}$, and the product of disease status for PPD coded as 0 or 1 by cell type proportions $(PPD \times P_{c})$. The model allows for covariates (not shown) and residual effects $E$. The cell proportions were estimated from bulk (cellularly heterogenous) DNA methylation data using available reference panels^16^. Coefficient $m_{c}$ is the effect of cell type $c$. The case-control difference $m_{c}^{PPD}$ for cell type $c$ is used to test the null hypothesis that cell type means are equal for cases and controls. Note that the model has no constant due to $\sum_{c=1}^{n_{c}} P_{c}\cong1$. Alternatively, the model is sometimes written with a constant whereby one of the cell type proportions is omitted ^21^ but this produces identical results ^19,22,23^.

## **REFERENCES**

1. Guintivano J, Sullivan PF, Stuebe AM, et al. Adverse life events, psychiatric history, and biological predictors of postpartum depression in an ethnically diverse sample of postpartum women. *Psychol Med.* 2017:1-14.

2. Cox JL, Holden JM, Sagovsky R. Detection of postnatal depression. Development of the 10-item Edinburgh Postnatal Depression Scale. *Br J Psychiatry.* 1987;150:782-786.

3. Otsubo T, Tanaka K, Koda R, et al. Reliability and validity of Japanese version of the Mini-International Neuropsychiatric Interview. *Psychiatry Clin Neurosci.* 2005;59(5):517-526.

4. Sheehan DV, Lecrubier Y, Sheehan KH, et al. The Mini-International Neuropsychiatric Interview (M.I.N.I.): the development and validation of a structured diagnostic psychiatric interview for DSM-IV and ICD-10. *J Clin Psychiatry.* 1998;59 Suppl 20:22-33;quiz 34-57.

5. Sahraeian SME, Mohiyuddin M, Sebra R, et al. Gaining comprehensive biological insight into the transcriptome by performing a broad-spectrum RNA-seq analysis. *Nat Commun.* 2017;8(1):59.

6. Yates A, Akanni W, Amode MR, et al. Ensembl 2016. *Nucleic Acids Res.* 2016;44(D1):D710-716.

7. Guintivano J, Shabalin AA, Chan RF, et al. Test-statistic inflation in methylome-wide association studies. *Epigenetics.* 2020;15(11):1163-1166.

8. Aryee MJ, Jaffe AE, Corrada-Bravo H, et al. Minfi: a flexible and comprehensive Bioconductor package for the analysis of Infinium DNA methylation microarrays. *Bioinformatics.* 2014;30(10):1363-1369.

9. Chen YA, Lemire M, Choufani S, et al. Discovery of cross-reactive probes and polymorphic CpGs in the Illumina Infinium HumanMethylation450 microarray. *Epigenetics.* 2013;8(2):203-209.

10. Teschendorff AE, Marabita F, Lechner M, et al. A beta-mixture quantile normalization method for correcting probe design bias in Illumina Infinium 450 k DNA methylation data. *Bioinformatics.* 2013;29(2):189-196.

11. Shabalin AA, Hattab MW, Clark SL, et al. RaMWAS: fast methylome-wide association study pipeline for enrichment platforms. *Bioinformatics.* 2018;34(13):2283-2285.

12. Pidsley R, CC YW, Volta M, Lunnon K, Mill J, Schalkwyk LC. A data-driven approach to preprocessing Illumina 450K methylation array data. *BMC Genomics.* 2013;14:293.

13. Elliott HR, Tillin T, McArdle WL, et al. Differences in smoking associated DNA methylation patterns in South Asians and Europeans. *Clin Epigenetics.* 2014;6(1):4.

14. Zeilinger S, Kuhnel B, Klopp N, et al. Tobacco smoking leads to extensive genome-wide changes in DNA methylation. *PLoS One.* 2013;8(5):e63812.

15. Cabrera CP, Navarro P, Huffman JE, et al. Uncovering networks from genome-wide association studies via circular genomic permutation. *G3 (Bethesda).* 2012;2(9):1067-1075.

16. Houseman EA, Accomando WP, Koestler DC, et al. DNA methylation arrays as surrogate measures of cell mixture distribution. *BMC Bioinformatics.* 2012;13:86.

17. Koestler DC, Christensen B, Karagas MR, et al. Blood-based profiles of DNA methylation predict the underlying distribution of cell types: a validation analysis. *Epigenetics.* 2013;8(8):816-826.

18. Venet D, Pecasse F, Maenhaut C, Bersini H. Separation of samples into their constituents using gene expression data. *Bioinformatics.* 2001;17 Suppl 1:S279-287.

19. Shen-Orr SS, Tibshirani R, Khatri P, et al. Cell type-specific gene expression differences in complex tissues. *Nat Methods.* 2010;7(4):287-289.

20. Donovan MKR, D'Antonio-Chronowska A, D'Antonio M, Frazer KA. Cellular deconvolution of GTEx tissues powers discovery of disease and cell-type associated regulatory variants. *Nat Commun.* 2020;11(1):955.

21. Montano CM, Irizarry RA, Kaufmann WE, et al. Measuring cell-type specific differential methylation in human brain tissue. *Genome Biol.* 2013;14(8):R94.

22. Chan RF, Turecki G, Shabalin AA, et al. Cell Type-Specific Methylome-wide Association Studies Implicate Neurotrophin and Innate Immune Signaling in Major Depressive Disorder. *Biol Psychiatry.* 2020;87(5):431-442.

23. Zheng SC, Breeze CE, Beck S, Teschendorff AE. Identification of differentially methylated cell types in epigenome-wide association studies. *Nat Methods.* 2018;15(12):1059-1066.
