# Supplementary figures and images for: Transcriptome-wide association study for postpartum depression implicates altered B-cell activation and insulin resistance
Source: Mol Psychiatry. 2022 Apr 1;27(6):2858–67. doi: 10.1038/s41380-022-01525-7 (PMC9156403; doi:10.1038/s41380-022-01525-7)

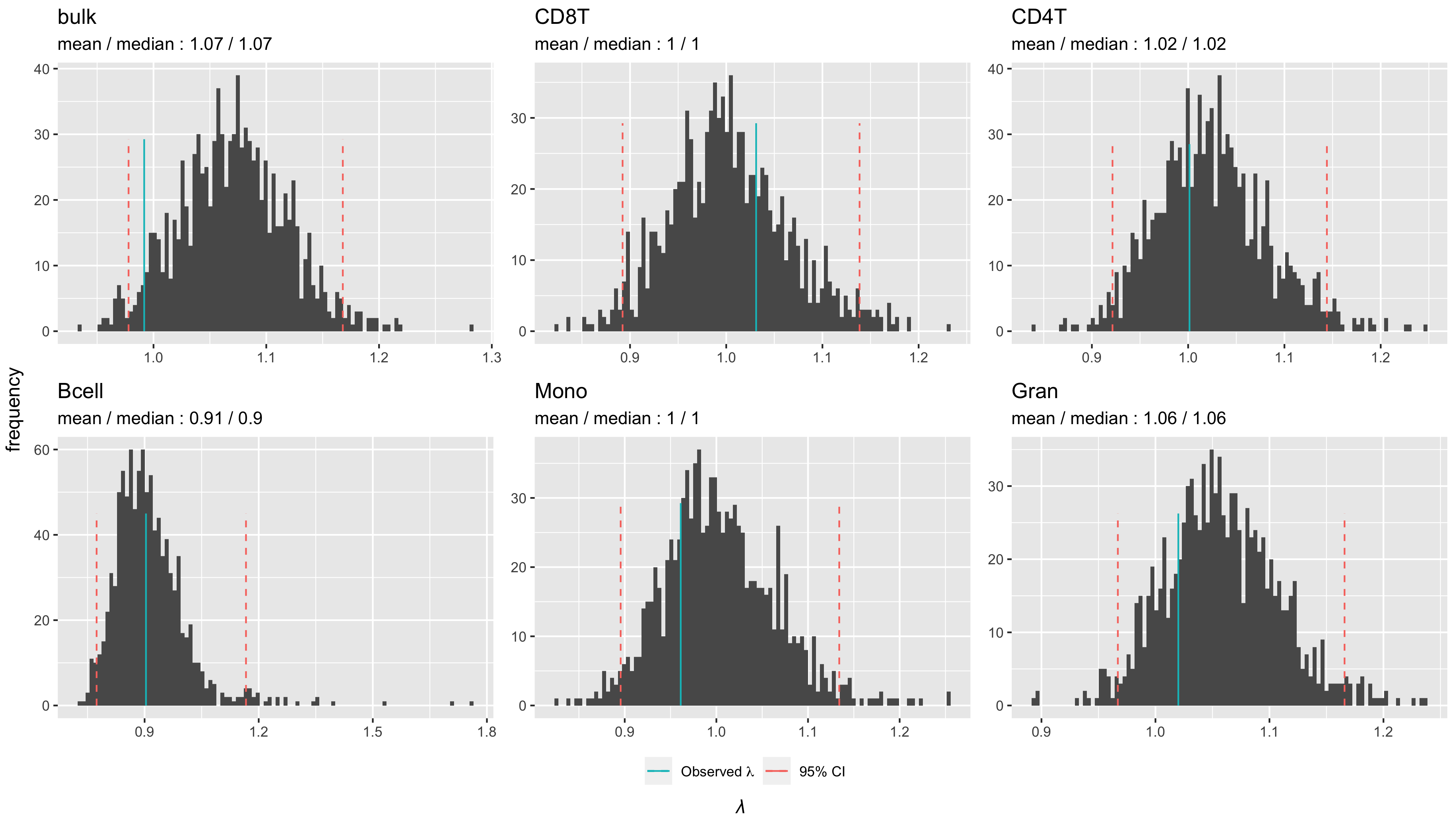

Supplement: Supplementary file 2 — Figure S1 [file 41380_2022_1525_MOESM2_ESM.png]

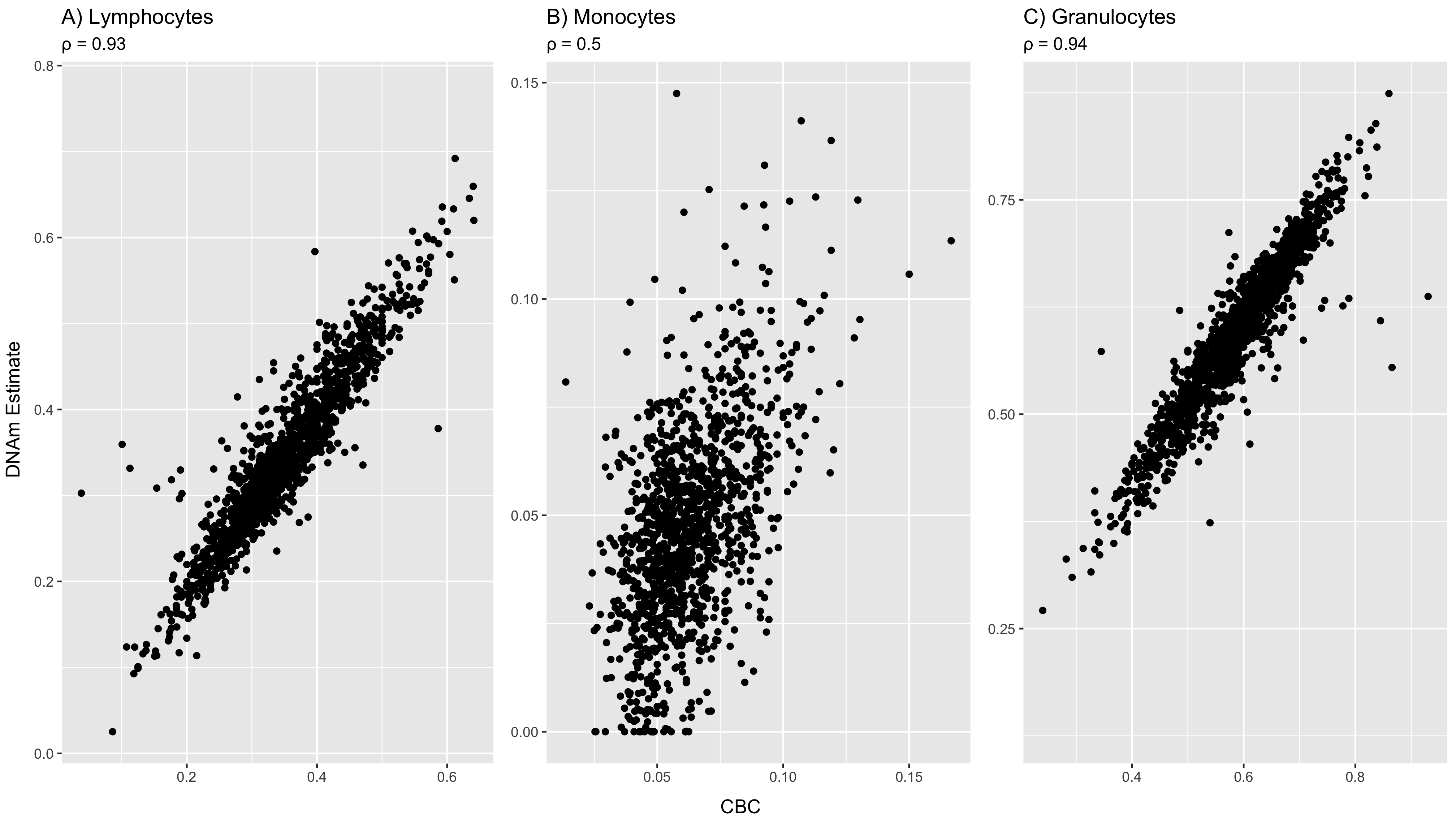

Supplement: Supplementary file 3 — Figure S2 [file 41380_2022_1525_MOESM3_ESM.png]

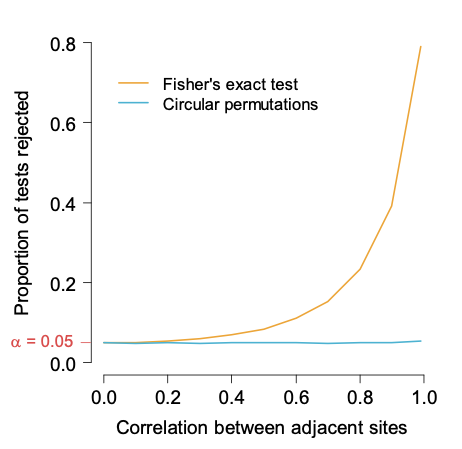

Supplement: Supplementary file 4 — Figure S3 [file 41380_2022_1525_MOESM4_ESM.png]

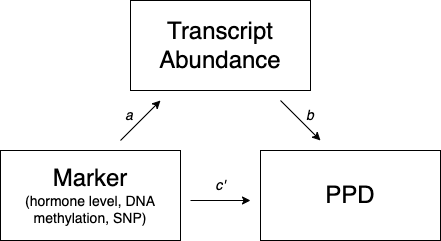

Supplement: Supplementary file 5 — Figure S4 [file 41380_2022_1525_MOESM5_ESM.png]
